# Parliament and the Profession

**Published:** 1917-11-03

**Authors:** 


### Parliament and the Profession.

### Imports of Opium.

MR. WARDLE, Parliamentary Secretary to the Board of Trade, in answer to an inquiry as to whether Indian opium is now replacing that of Turkish or Persian origin in the manufacture of morphia and official preparations, gave some statistics of the net imports of opium retained in this country. While that coming from Persia, Turkey, and other countries had fallen from 350,536 lb. in 1913 to 123,442 lb. in 1916, the imports from India in the same period had increased from 4,824 lb. to 437,237 lb.

### Hospital Treatment of Ophthalmia Neonatorum.

MR. HAYES FISHER informed Sir William Collins that the Local Government Board has pressed upon local authorities the importance of arranging for hospital beds for infants suffering from ophthalmia neonatorum, along with their mothers. Several larger local authorities have made arrangements, and the Metropolitan Asylums Board are prepared to provide two hospitals, one to serve the North of London and one the South.

### Medical Referees for Disabled Soldiers.

MR. PRATT, on behalf of the Pensions Minister, stated that in response to advertisements a number of applications for the post of medical referee have been received, and the qualifications of the candidates are being considered by a Committee of Selection, consisting of representatives of the Central Medical War Committee, the Local Government Board, and the Insurance Commission. Over a hundred medical referees, covering all the Southern, and some of the South Midland Counties of England, and the whole of Wales, have been appointed. About 300 further appointments will be made.

### Sphagnum Moss—Cost of Carriage.

THE Parliamentary Secretary to the Board of Trade, in answer to a question, said that the Railway Executive Committee have considered applications for the free conveyance of sphagnum moss, but it has not been found possible to grant exceptional treatment to this traffic.

